# Unilateral Focal Retinitis as an Initial Manifestation of Cat-Scratch Disease

**DOI:** 10.7759/cureus.30907

**Published:** 2022-10-31

**Authors:** David F Santos, Sofía C Ayala Rodríguez, Guillermo A Requejo Figueroa, Mariella Pappaterra-Rodriguez, Armando L Oliver

**Affiliations:** 1 Ophthalmology, University of Puerto Rico School of Medicine, Medical Sciences Campus, San Juan, USA

**Keywords:** case report, azythromycin, cat-scratch disease, focal retinitis, bartonella henselae

## Abstract

We report on a case of focal retinitis as the initial manifestation of cat-scratch disease. A 56-year-old Hispanic woman presented for a routine follow-up examination. A fundus examination of the right eye revealed a white retinal lesion along the inferotemporal artery; this lesion was noted to have progressed after one week of observation. On further inquiry, the patient reported she had seven cats at home, some of which were less than six months old and had recently scratched her. She received empirical treatment for focal retinitis with azithromycin (500 mg daily) and valacyclovir (1 g three times daily), which would cover the most common parasitic, viral, and bacterial etiologies. She was lost to a follow-up examination. However, she continued the same dose of antibiotic and antiviral treatment. Upon her eventual follow-up, three months later, it was noted that the lesion had resolved. The initial work-up revealed that she was positive for *Bartonella henselae *IgM (1:20) and IgG (1:512), as well as for *B. quintana* IgG (1:256); however, she was negative for *B.* *quintana* IgM. At a four-month follow-up appointment, the *B. henselae* IgM was negative, the IgG had decreased from 1:512 to 1:64, and the *B. quintana* antibody test was negative for IgM and IgG, all of which are consistent with an adequately treated case of cat-scratch disease. Focal retinitis can be a rare initial manifestation of cat-scratch disease, which should be considered part of the differential diagnosis in cases of focal retinitis, especially in patients with a history of close contact with young cats. Additionally, oral azithromycin may be considered as a treatment for some cases of cat-scratch-associated focal retinitis.

## Introduction

Cat-scratch disease (CSD), caused by *Bartonella henselae *(*B. henselae*), a gram-negative, rod-shaped bacterium, is usually a self-limiting infection; it is characterized by fever, skin lesions, and regional lymphadenopathy [1]. The primary reservoir for *B. henselae* is cats [1]. Contact with cats that are under one year of age is documented in about 90% to 95% of CSD cases, and about 73% of these patients report having sustained a cat scratch [1].

In rare cases, CSD can manifest as a disseminated disease, with approximately 5% to 10% of patients demonstrating ocular symptoms [2]. Ocular involvement can be due to hand-to-eye contact, hematogenous spread, or the aerosol transmission of cat flea feces [1,3]. Such manifestations include Parinaud oculoglandular syndrome, neuroretinitis, intermediate uveitis, anterior uveitis, branch retinal vessel occlusion, and focal retinitis, among others [2].

While ocular manifestations of CSD have been described, the literature on focal retinitis due to *B. henselae* infection remains minimal. Therefore, we report on a case of focal retinitis of the posterior pole with a white retinal lesion as an ocular manifestation of CSD, including a review of the clinical course, laboratory work-up, photographic findings, and management.

This article was previously presented as a conference poster at the 2022 Annual Research and Education Forum on March 30, 2022.

## Case presentation

A 56-year-old Hispanic woman presented for a routine follow-up examination with a chief complaint of blurry vision in her right eye. The patient had a past medical history of hypertension, hypothyroidism, arthritis, fibromyalgia, and depression. She also had a history of pituitary adenoma with monocular diplopia. The patient had seven cats at home, including some that were less than six months old and had scratched her. The family history was remarkable for diabetes, hypertension, and heart disease. She denied any history of smoking, alcohol consumption, or illicit drug use, and the review of symptoms was unremarkable.

**Figure 1:**
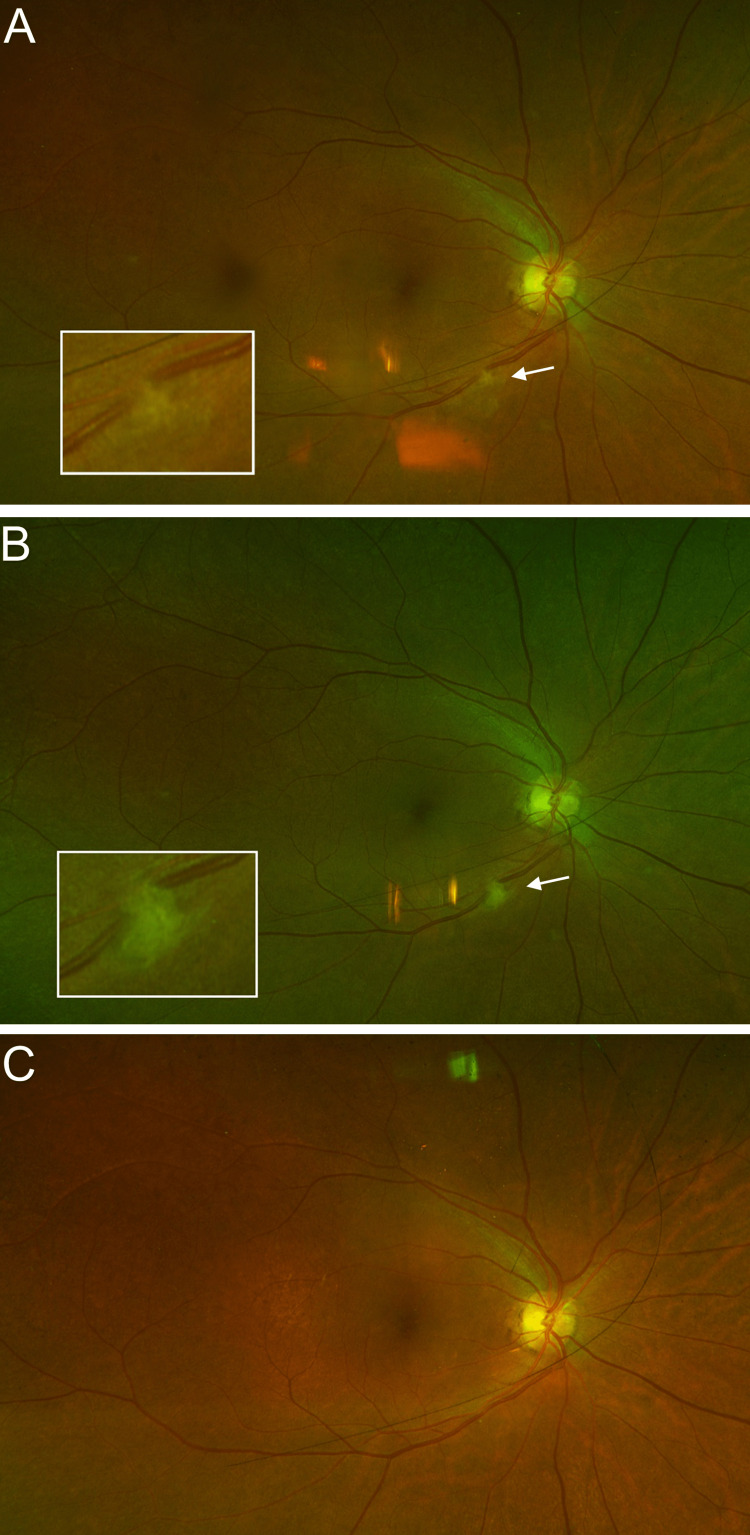
Color photograph of the right posterior pole. (A) At the initial examination, a small, white lesion (arrow) (see inset for image magnified three times) was noted along the inferotemporal arcade. (B) After one week of observation, the lesion (arrow) increased in size (see inset for image magnified three times), leading to a diagnosis of focal retinitis. (C) The lesion resolved following a course of systemic therapy with azithromycin.

**Figure 2:**
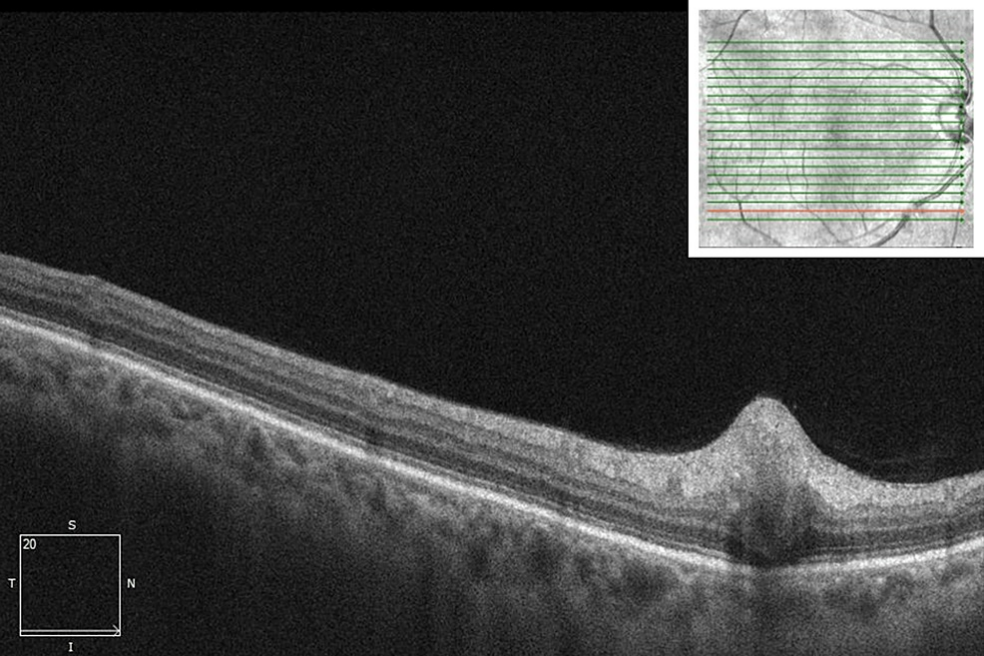
Optical coherence tomography (OCT) examination of the right macula. The analysis reveals a hyper-reflective retinal nerve fiber layer thickening in the area corresponding to the abnormal retinal lesion.

Upon a comprehensive examination, her best-corrected visual acuity was found to be 20/30+1 in the right eye and 20/40 in the left eye. The intraocular pressure was 9 mmHg in both eyes. The pupils were both round and reactive to light, without any relative afferent defect. Slit-lamp examination was unremarkable. The right fundus revealed a small area of retinitis along the inferotemporal arcade (Figure 1A), which increased in size after one week of observation (Figure 1B). Optical coherence tomography (OCT) examination of the lesion revealed associated retinal nerve fiber layer thickening (Figure 2).

An assessment of focal retinitis was made; therefore, the patient was treated empirically with oral azithromycin (500 mg daily) and valacyclovir (1 g three times daily). A work-up revealed her to be positive for *B. henselae* IgM (1:20) and IgG (1:512) as well as for *Bartonella quintana* IgG (1:256). The patient was lost to follow-up for three months, during which she continued to take oral azithromycin and valacyclovir and after which time her lesion had completely resolved (Figure 1C). Additional results revealed her to be positive for varicella zoster virus IgG, toxoplasma IgG, and herpes simplex virus 1 IgG. However, at a four-month follow-up appointment, the *B. henselae* IgM was negative and the IgG had decreased from 1:512 to 1:64. Moreover, the* B. quintana* antibody test was negative for IgM and IgG, all of which is consistent with an adequately treated case of CSD.

## Discussion

A small, fast-growing white retinal lesion with diffuse borders should alert the clinician to the possibility of focal retinitis [4]. The most common cause of focal retinitis is *Toxoplasma gondii* infection; however, other less common causes should be kept in the differential and ruled out according to the patient’s history and review of systems. Such causes include *Herpesviridae*, CSD, and *Cytomegalovirus* [5]. Empirical therapy should be considered for most cases, as the progression of focal retinitis may cause permanent visual loss if the fovea or optic disk becomes involved [5,6].

A diagnosis of CSD is based on having a recent history of contact with a cat, the current clinical features, and a positive serology defined as IgM positivity or IgG titers higher than 1:256 [7]. In this case, the patient’s history of having several cats at home, including young cats, and having been scratched led to the suspicion of CSD. However, as the disease is difficult to differentiate from necrotizing herpetic retinitis (based only on clinical examination findings), an empirical therapy was chosen that would include the most common pathogens, such as toxoplasma, herpes, and *Bartonella*. Once the initial work-up revealed positive *B. henselae* IgM (1:20) and IgG (1:512), the diagnosis of CSD was confirmed.

Several clinical factors can assist in the diagnosis of CSD-associated retinitis, including the patient’s age, the time of contact with a cat, the systemic symptoms, the manifestations of *Bartonella* infection, and host immunity [8,9]. Other nonspecific symptoms include conjunctivitis, hepatosplenic disease, and neurologic manifestations, such as neuritis and encephalitis [10]. Intraocularly, CSD most commonly presents as a neuroretinitis, the most common posterior segment complication of CSD [7,11,12]. In most cases, neuroretinitis develops two to three weeks following the initiation of systemic symptoms such as arthromyalgia [12]. It commonly presents with reduced visual acuity, optic disc inflammation, and partial or complete exudates around the macula in one eye [7-10]. Studies have also reported isolated foci of retinitis or choroiditis as common ocular manifestations of CSD [13]. Unilateral involvement is far more common than bilateral involvement [14].

Due to the self-limiting nature of CSD and the current lack of randomized controlled trials, guidelines for ocular and systemic CSD treatment are incomplete [8,9]. Several studies have indicated that ocular inflammation is reduced and that immediate visual recovery occurs when antibiotic therapy is initiated within a few days of the onset of symptoms [10]. The most frequently used antibiotic in adults is doxycycline, which is usually prescribed at 100 mg twice a day for two to four weeks in immunocompetent individuals and up to four months in immunocompromised patients [7]. Azithromycin has also been demonstrated to be an effective option for the treatment of CSD. Compared to doxycycline, azithromycin has the advantage of requiring less-frequent dosing and of having an increased safety profile [15]. Some authors believe the visual outcome may be improved with the use of oral corticosteroids in addition to antibiotics [9,14].

## Conclusions

Unilateral focal retinitis may be a rare initial manifestation of CSD and can be indicative of disseminated *B. henselae* infection. It should be considered as part of the differential diagnosis, particularly in patients with a history of cat contact, as it may be the only symptom of CSD. Our case suggests oral azithromycin may be an effective treatment for focal retinitis due to ocular CSD. Ocular manifestations of CSD should be investigated further in future studies to improve patient diagnosis and reduce long-term impairments.
